# MULTI-OMICS ANALYSIS IDENTIFIES GENE NETWORKS ASSOCIATED WITH COGNITIVE AGING AND ALZHEIMER’S DISEASE

**DOI:** 10.1093/geroni/igz038.2179

**Published:** 2019-11-08

**Authors:** Catherine Kaczorowski, Sarah Heuer, Chris Gaiteri, Catherine Kaczorowski

**Affiliations:** 1 The Jackson Laboratory, Bar Harbor, Maine, United States; 2 Rush University, Chicago, Illinois, United States

## Abstract

Alzheimer’s disease (AD) is the leading cause of age-related dementia, yet no treatment exists. AD is heterogeneous, and is resultant of the dysregulation of many genetic and biological processes. To decipher this complexity, we leveraged the first translational mouse population of AD to identify 15 gene networks related to individual differences in cognitive outcomes. Using QTL mapping, we also identified a novel putative driver of a module, Gstk1, highly conserved in humans that also significantly correlated with memory outcomes. Together, these transcriptional networks provide new mechanistic insight into the biological processes that regulate individual differences in cognitive function across a genetically diverse population. We could identify how demographics (age, sex, causal AD mutations) influence these modules and how they relate to cognitive outcomes. Finally, the high degree of conservation between our mouse modules to human modules reflects the translatability of our model to human AD, adding to its face validity.

